# Computational Challenges of Personal Genomics

**DOI:** 10.2174/138920208784139564

**Published:** 2008-04

**Authors:** Hamid Bolouri

**Affiliations:** Division of Biology, California Institute of Technology, CA 91125, USA

**Keywords:** Genomics, personal genomes, personalized medicine, predictive and preventive medicine, gene expression variability.

## Abstract

It is widely predicted that cost and efficiency gains in sequencing will usher in an era of personal genomics and personalized, predictive, preventive, and participatory medicine within a decade. I review the computational challenges ahead and propose general and specific directions for research and development. There is an urgent need to develop semantic ontologies that span genomics, molecular systems biology, and medical data. Although the development of such ontologies would be costly and difficult, the benefits will far outweigh the costs. I argue that availability of such ontologies would allow a revolution in web-services for personal genomics and medicine.

## INTRODUCTION

The cost per nucleotide of DNA sequencing has been dropping exponentially for more than a decade [1]. In 2005, the National Human Genome Research Institute (NHGRI) awarded a series of research grants to develop technologies capable of sequencing an entire human genome for $1000. Currently, there are at least nine commercial ventures racing towards this goal [1].

While the availability of cheap, diploid, full-genome sequences may still be several years away, low-cost tests for large numbers of SNPs and other sequence variations are already being used by companies such as 23andMe (https://www.23andme.com/), NaviGenetics (http://www.navigenics.com/) and deCODE Genetics (http://www.decode.com/) to provide personal disease-susceptibility profiles. Other companies, such as KNOME (http://www.knome.com/) are offering full personal genome sequencing for those who can afford the current costs. 

Thus, DNA sequence variability data is becoming increasingly available to individuals. The extent to which the availability of such data will lead to improvements in health-care depends on four criteria, as set out by the US Centers for Disease Control (http://www.cdc.gov/genomics/gtesting/ACCE.htm):

### 1 Accuracy of genotyping

The frequency of incorrect calls must be very low indeed before personal genetic profiles can be commercially viable. The accuracy will depend on the sequencing technology. Due to factors such as fold-coverage, it will to some extent be determined by economic considerations.

### 2 Predictive value of genotypes

Assuming a list of correctly identified genetic polymorphisms in an individual, the extent to which we can predict phenotypic outcomes will vary according to how much is known about the identified polymorphisms, the extent to which environmental and life-history factors can influence the phenotypes, etc.

### 3 Clinical utility of knowing genotypes

Even given an accurate genotype and a high-confidence prediction of a phenotype, we may not be able to predict the rate of deterioration, or the risk of unacceptable adverse-effects for some treatments, or a treatment may simply not be available. For these and many other reasons, it is not always useful to know disease-associated genotypes.

### 4 Ethical, legal and social issues arising

Considerable concerns have been raised over the lack of standards for genetic test, information security, potential social stigma/discrimination, and provision of impartial advice to ‘patients’ (see for example [2]). Though government regulations such as the US Genetic Information Non Discrimination Act [3] have been discussed for over a decade, public awareness and debate on these issues is still limited to specialist communities.

In this paper, I will assume that genotype data made available to individuals will be very high quality, and doubly tested by secondary means to ensure against false calls. Going further, let us assume that we have:

- No legal (e.g. government laws) or commercial (e.g. patent restrictions and discriminatory insurance practices) barriers to personal genomics tests.
- Many examples of perfectly sequenced full diploid genomes as publicly available points of reference (e.g. through the Personal Genome Project (http://www.personalgenomes.org/).
- Increasingly detailed and comprehensive genetic data through public efforts such as the 1000 Genomes Project (http://www.1000genomes.org/).
- Perfect knowledge of all genetic variants in an individual (i.e. 100% coverage of every polymorphism in the individual).
- Detailed and comprehensive assays that characterize an individual’s life-history, environmental health factors, and current health status.

The question posed in the remainder of this paper is – given the above assumptions – what computational resources will we need to

- Allow physicians and ‘patients’ to predict an individual’s disease susceptibilities?
- Evaluate the impact of life-history and life-style choices on these susceptibilities.
- Monitor potential progression towards predicted disease phenotypes.
- Recommend preventive measures (e.g. life-style changes, or surgery).
- Critique treatment alternatives (e.g. drugs vs. surgery).
- Learn from experience with one patient and apply the lesson to other cases.
- Keep patients’ data confidential, but allow access for research purposes.

## GENETIC VARIABILITY IN HUMANS

The prevalence of DNA sequence polymorphisms within the human population has been long-studied and well-documented. Recent mapping studies [4, 5] and analysis of the genomic sequences of Craig Venter [6] and James Watson (http://jimwatsonsequence.cshl.edu/), have confirmed the prevalence of both small-scale (<20bp) and large-scale (thousands of base pairs long) polymorphisms in individuals. For example, one or more of the following polymorphisms affect 44% of Venter’s genes: 3,213,401 SNPs, 53,823 block substitutions (2–206 bp), 292,102 heterozygous indels (1–571bp), 559,473 homozygous indels (1–82,711bp), and 90 inversions [6].

Traditionally, studies of DNA polymorphisms have focused on direct associations with disease (for a recent review, see [7]). For example, the Online Mendelian Inheritance in Man (OMIM) database (http://www.ncbi.nlm.nih.gov/omim/) currently lists 18,440 phenotype-associated polymorphisms, while the Human Gene Mutation (HMG) database (http://www.hgmd.cf.ac.uk/) currently houses over 76,000 disease-associated genetic mutations (including small and large indels). 

Recent studies suggest that many polymorphisms not associated with specific diseases may be responsible for significant differences in gene expression levels between ‘normal’ ‘healthy’ individuals (see below). Indeed, it now seems likely that evolutionary forces such as Balancing Selection [8,9] are maintaining a diversity of gene expression levels among individuals. One implication of these findings is that healthy individuals may respond differently to the same genotype depending on their genomic background. Another implication is that different individuals may respond to the same drug very differently. Below, we explore these issues further.

## GENE EXPRESSION VARIABILITY AMONG INDIVIDUALS

Gene expression variation in humans is known to arise from at least three different mechanisms. First, non-genetic factors such as age, gender, and health history are well known to affect gene expression. Metabolic status [10], smoking [11], exercise [12], and emotional state [13] have also been shown to modulate the expression of large numbers of genes by 2-fold or more. For example, regular exercise has been shown to lead to increased levels of the immune system related cytokine IL-6 in muscle cells and also in the blood [14]. Such effects clearly have health implications.

Second, inherent transcriptional noise in individual cells arises from random variations in cellular content, and the fact that transcription is a sequential process involving a small number of copies of each gene [15]. Measurements in single mammalian cells suggest a protein abundance coefficient-of-variation of 15-30%, and long-lasting (>24 hours) concentration changes [16]. Thus, the abundance of the same protein in two genetically identical cells at steady state may be two to four fold different (mean+/-2SD) purely due to gene expression noise. 

Fig. (**1**) shows simulated mRNA abundance distribution in 1000 genetically identical cells for a partially repressed gene. The regulatory scenario is depicted in the inset. A repressor R and an activator A compete to set the transcriptional activity level of the gene. Both A and R protein abundances are assumed to be distributed Normally in the 1000 cells. The fractional promoter occupancy of the gene is therefore modeled as: 

$\mathrm{occupancy}=\frac{K_{A}.\left[A\right]}{1+K_{A}.\left[A\right]+K_{R}.\left[R\right]}$

where square brackets denote concentrations, and K’s represent the respective protein-DNA binding association constants. When [R]>0 and occupancy>0, the occupancy of the gene will be approximately the ratio of two Normal distributions, and therefore long-tailed. This characteristic is clearly visible in Fig. (**1**). Compare the histogram distribution of mRNA molecules per cell to the super-imposed Normal distribution with the same mean and variance (continuous curve).

Such long tails in gene expression levels have been widely observed (e.g. [17]). But their effect on susceptibility to disease and response to treatment is not yet clear. There is some evidence that some genetic regulatory networks employ feedback loops to reduce the degree of gene expression heterogeneity among cells [18]. At present, it is not clear how wide-spread such noise control mechanisms may be.

Overall, the above two mechanisms can each cause in the order of 2-fold expression-level differences between two genetically identical individuals. The third and largest cause of gene expression variability is DNA sequence polymorphisms. Polymorphisms within and across populations have been widely reported to cause many-fold differences in gene expression among individuals [19-21]. Remarkably, variations within racial groups appear to be of the same order of magnitude as variations between the averages for different racial groups. While expression differences as high as 200-fold have been reported for specific genes [20], the average variability appears to be in the order of 10-fold. This level of variation is observed in multiple cell types and for a large proportion of genes in both healthy and diseased individuals [21]. Genes involved in the immune system – a target of many drugs – appear to be particularly variable, both within and among populations [22]. 

At present, it is not clear whether the above three sources of gene expression variability are additive in effect. But even if they are not, it appears that for a majority of genes, gene expression differences of ten fold or more may be common between any pair of individuals.

## THE NEED FOR PERSONALIZED MEDICINE

The effect of sequence polymorphisms on drug uptake and clearance rates has been well-studied over the past decade (see for example [23]). Polymorphisms in certain genes such as the Cytochrome P450 family can lead to considerable variation in drug dose response. For example, the dosage requirement for the anti-coagulant drug warfarin can vary by as much as 20-fold depending on polymorphisms in at least 24 DNA loci [24].

Given that gene expression variability among individuals is widespread and large in magnitude (see preceding section), there will be significant differences in the way individuals respond to specific disease-associated mutations, environmental health insults (e.g. pathogens, toxic substances), and treatment regimes. For example, protective mutations against health threats as different as the HIV virus [25] and tobacco addiction [26] are well documented. 

It is therefore increasingly clear that effective diagnosis (identification of danger signs), prognosis (forecast of disease progression), and treatment plans (selection of an appropriate course among multiple alternatives) must be tailored to the specific genetic and health-profile of each individual patient.

## THE NEED FOR PREDICTIVE (NETWORK) MODELING OF PERSONAL DATA

The recent sequencing and analysis of Craig Venter’s full diploid genome [6] has revealed a surprisingly large number of multiple ploymorphisms affecting shared pathways in a single person. For example, Venter’s genome includes multiple genotypes associated with the increased likelihood of tobacco addiction. However, an additional polymorphism provides protection against tobacco addiction.

Another example of potentially complex interactions among multiple allelic variants arises from Venter’s genotypes for susceptibility to Alzheimer’s Disease (AD). Disease-associated alleles for *sorl1* and *apoE* are reported. Fig. (**2**) shows the KEGG (http://www.genome.jp/kegg/) pathway diagram for AD. Note the close interaction of the reported polymorphisms with the Amyloid Precursor Protein (APP). A third molecule, lipoprotein lipase (LPL) also has a common AD-associated polymorphism and interacts closely with the preceding three molecules. 

To predict the subject’s susceptibility to AD, we will need to understand the outcome of these and other nonlinear interactions within the context of other variabilities within the genome and the life/health history of this particular individual. Predictive modeling for individual patients is a far more challenging concept than anything currently attempted.

## THE NEED FOR PARTICIPATORY MEDICINE

As the complexity of diagnoses and treatment options increases, physicians are increasingly put in the difficult position of (A) having to explain the data and their implications to patients, and (B) having to make recommendations on the basis of probabilistic predictions. One way to address these issues is to empower *participatory* medicine, i.e. encourage patients to take an active role in healthcare planning, and making informed choices about preventive medicine. 

Given that the data and their interpretation is so complex, how can we equip the public with intuitive analysis and visualization tools that allow them to understand the interpretations of their data, explore prognostic models, and compare treatment alternatives? Current patient-oriented educational and analytic resources such as internet searches [27], blogs (e.g. http://mydaughtersdna.org/) and simple risk assessment calculators (see for example, http://www.framing hamheartstudy.org/risk/hrdcoronary.html) will be woefully inadequate in the era of personal genomes. 

A complicating factor with respect to the patient-physician relationship is known as the Expert Services Problem [28]. Diagnosis is becoming increasingly costly and complex, and there are often multiple treatment options with differing trade-offs. As there is little comparability among patients, health service customers cannot ‘compare notes’. Moreover, diagnosis and treatment are usually performed by the same health care provider (who may be subject to financial incentives and advertising ‘spin’ from the pharma and biotech industries). Thus, post-hoc realization of non-optimal diagnoses or treatment may not be revealed to patients.

In short, patients increasingly find it difficult to judge the quality and impartiality of the diagnosis and treatment recommendations they receive. This leads to a moral hazard: health providers can profit from ‘over-treating’. This is clearly an undesirable situation. In the long term, both patients and health providers will suffer if healthcare providers feel there is little reward for better quality of work. These concerns can also be alleviated through empowering patients to understand their health status and prognosis better.

## THE NEED FOR INFORMATION AGGREGATORS AND FILTERS

Fig. (**3**) shows the number of research papers published on six common diseases over the period January 2000 – January 2007. Even for ‘less studied’ diseases such as Osteoarthritis and Malaria, the number of papers published per day exceeds the reading time available to any individual. Moreover, relatively few individuals will have the technical knowledge to understand the subject matter and judge the implications of the findings appropriately. 

In response to the above information glut, much recent research has focused on developing information aggregators. For example, the pathway resources directory PathGuide (http://www.pathguide.org/) currently lists 240 databases of biological pathways. It is very useful to have a central directory of all available pathway databases. But it is unlikely that anyone will have the time and expertise to retrieve relevant content from all 240 available databases, judge their relevance, and extract useful conclusions. 

There is clearly a need for computational approaches that summarize relevant and trustworthy data from the very large amount of information available in publications and databases, and make them available to researchers, practicing physicians, and proactive patients in easily digestible forms.

## COMPUTATIONAL SUPPORT FOR PERSONAL GENOMICS AND PERSONAL MEDICINE

The preceding sections focused on describing some of the computational challenges posed by personal genomics. The purpose of this section is to suggest some possible solutions. In particular, I propose that existing internet-based technologies can be adapted to meet most of the challenges envisaged. 

Consider the following example “use case scenarios”:

1. An individual has had multiple genomic, proteomic and other analysis performed, and now needs to understand the implications of the results. Should she have an operation (potentially debilitating, or dangerous); should she take a drug (with inevitable side-effects); or should she make (potentially difficult or costly) "pre-emptive" life-style changes?
2. A physician has received the above person's data and is considering the same questions. The physician requires greater technical details and more quantitative data (e.g. serum test data, drug dose-response/interaction data, etc), and additional information on legal and contractual constraints (e.g. FDA regulations, and patient insurance terms), but she may not have extensive training in genetics, and will not have time to read the vast majority of the research literature relevant to the patient’s data.
3. A research scientist is working on a disease to which the above patient/client appears to be susceptible. The researcher can pay far greater attention to detail than either the ‘patient’ or the physician. She will also be willing and able to use much more sophisticated data analysis tools, and may well spend many years investigating the same topic and building up personal familiarity with the subject.

The patient, doctor and researcher all need to understand the same pool of available data, and share many challenges (data overload, too many tools to learn, complex theory underlying analysis methods, etc.). The conjunction of the interests of these three large user-communities provides a very large potential market, and makes the development of a computational infrastructure for personal genomics an attractive investment.

### Semantic Ontologies for Personal Genomics

The development of a semantic ontology (the association of meaning with data) for personal genomics and health informatics would be a crucial first step to the development of computational resources for personal genomics.

While the development of a general-purpose ‘semantic web’ may be too difficult to yield fruit in the near future, ontologies for well-defined scientific domains such as medical informatics and genomics are much easier to define unambiguously. Indeed, efforts to develop a semantic ontology for medical informatics are well underway (http://esw.w3.org/topic/SemanticWebForLifeSciences). Similar efforts are needed for personal genomics. In particular, personal genomics will need to build a semantic bridge between the medical community, and the molecular systems biology community, which also has a number of emerging ontology standards (e.g. http://www.geneontology.org/, http://sbml.org/, http://www.cellml.org/,  http://www.biopax.org/). 

The establishment of a common semantic ontology for personal genomics and medicine would permit two important developments. First, it allows software tools to interpret genetic, genomic, pharmacological, and medical data. This in turn will allow the development of sophisticated computational tools that adapt their resources and output to the needs and expertise of their users. 

Second, and at least as important, semantically tagged personal genomic, pharmacological, and medical data permit ‘bottom-up’ (or community-based) participation in the analysis of public data. Semantically-tagged data can be computationally filtered, categorized, and summarized. As a result, users who are not experts in computer science, biology, or medicine can use web-services to search large numbers of semantically-tagged databases, and collate their own dossiers of relevant, up-to-date information.

Community-based internet resources such as customer ratings, reviews, and wikis have already had a major impact in many domains of life. They are characterized by their participatory and highly fluid content, and benefit from ‘wisdom of the crowds’ effects. These characteristics stand in direct contrast to existing ‘top-down’ (or authoritative) information resources (e.g. FDA databases), which typically enforce objectivity and hierarchical quality assurance at the expense of adaptability and rapid response. 

In recent years, both medical informatics systems and computational molecular systems biology have come of age. As discussed below, there are already a broad range of computational resources that could be adapted for use in personal genomics and personalized medicine settings.

### Medical Informatics Frameworks

In medical informatics, there are already a number of excellent medical information management systems. Two good examples are the award-winning VISTA system developed by the Veteran’s Association of USA (http://www.virec.research.va.gov/DataSourcesName/VISTA/VISTA.htm) and the clinical health services framework implemented by the Boston-based CareGroup [29]. The latter currently serves over 3000 MDs, handles some 250 Terra Bytes of data per day, and employs some 250 IT staff. Thus, by any measure, there is track-record and willingness in the medical sector for the introduction of strong computational support.

At present, the above medical informatics resources focus on providing automated patient records and transaction support (e.g. physician-physician consultations, ordering drugs, setting up treatment plans, etc). However, efforts are also under way to allow these frameworks to provide some decision support. For example, the system implemented by CareGroup alerts physicians if the patient is known to have an allergy to a drug they have prescribed. Thus, decision-support based on more detailed patient data would be a natural further development in medical informatics.

### Computational Systems Biology

In the area of molecular systems biology too, many useful tools have been developed for data visualization, analysis and modeling. These include tools for integrative network modeling (e.g. http://cytoscape.org/ and http://www.biotapestry.org/), tools for exploring genome-scale associations (e.g. http://www.caida.org/tools/visualization/walrus/), tools for concurrent, multiple transformations and visualizations of high-dimensional data (e.g. http://www.ggobi.org), selective-focus comparisons of hierarchical categorical data (http://olduvai.sourceforge.net/), and many more. My purpose in listing the preceding examples is to emphasize the breadth of tools available and their relative maturity.

At present, there is no coupling between the above molecular biology research tools and the clinical IT environments described earlier. Any information flow between the two domains is currently ad-hoc and primarily through individuals with interests in both domains. With the advent of personal genomics it is increasingly imperative that we integrate basic research, medical practice, and patient-centered services. 

Fig. (**4**) provides a schematic summary of such integration. Currently, data flow from research to practice occurs over years. In the proposed scheme, the computational infrastructure for these two communities is shared. One advantage of such sharing is that the tools that the patient, physician, and research communities use can essentially be different user-interfaces to the same underlying computational engines. Another advantage is that results from specific medical tests can be analyzed in the context of global datasets. The gray arrow going back from medicine to molecular biology research indicates that the proposed infrastructure will also permit researchers to explore clinical data. Such access can be mediated through security-certified web services, as outlined below. 

### Data Privacy and Security

Given that complex data analysis will need to be performed in order to interpret the health implications of DNA polymorphisms, how will we ensure the privacy of patients and the security of the data? In spite of the removal of personal identity information from public databases, it may be possible to reconstruct individual identities by association of multiple genetic traits and medical records with personal profiles (e.g. health histories) of individuals.

Ideally, we need to ensure complete patient privacy, while allowing anonymized patient data to be mined for research. One solution is to allow individuals to keep their own data, and to make informed choices as to who has access to the data. Researchers and medical practitioners can use web services and applications that do not store data locally (such as Java web start) to analyze data on-the-fly through pre-defined filters. By keeping data in only one location (i.e. the patient’s own data vault), data security risks can be minimized while maximizing data access privileges. 

## CONCLUSIONS

Personal genomics may not be profitable yet, but it is already a commercial reality. The extent to which personal genomics and personalized medicine will benefit the public at large will depend on the extent to which the technological breakthroughs are accompanied by appropriate computational developments that allow users to participate in determining appropriate responses to diagnostic data. Moreover, we need to develop the appropriate computational infrastructure before the technology for personal genomics becomes widely available, because poorly interpreted data is likely to lead to misdiagnoses and a potential public backlash against a very promising revolution in medicine.

## Figures and Tables

**Fig. (1):**
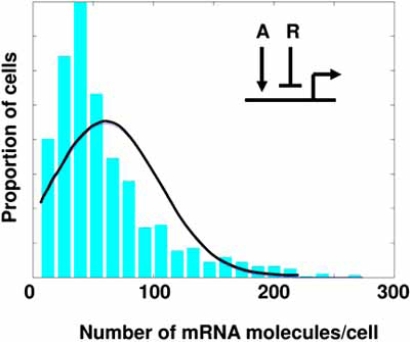
**Gene expression variability among genetically identical cells**. Simulated mRNA abundance distribution in 1000 genetically identical cells for a partially repressed gene. The histogram shows the distribution of mRNA molecules per cell. Super-imposed is a Normal distribution with the same mean and variance (continuous curve). The scale of the horizontal axis (mRNA abundance) is arbitrary. **Inset**: A repressor R and an activator A compete to set the transcriptional activity level of the gene. Both A and R protein abundances are assumed to be distributed Normally in the 1000 cells.

**Fig. (2):**
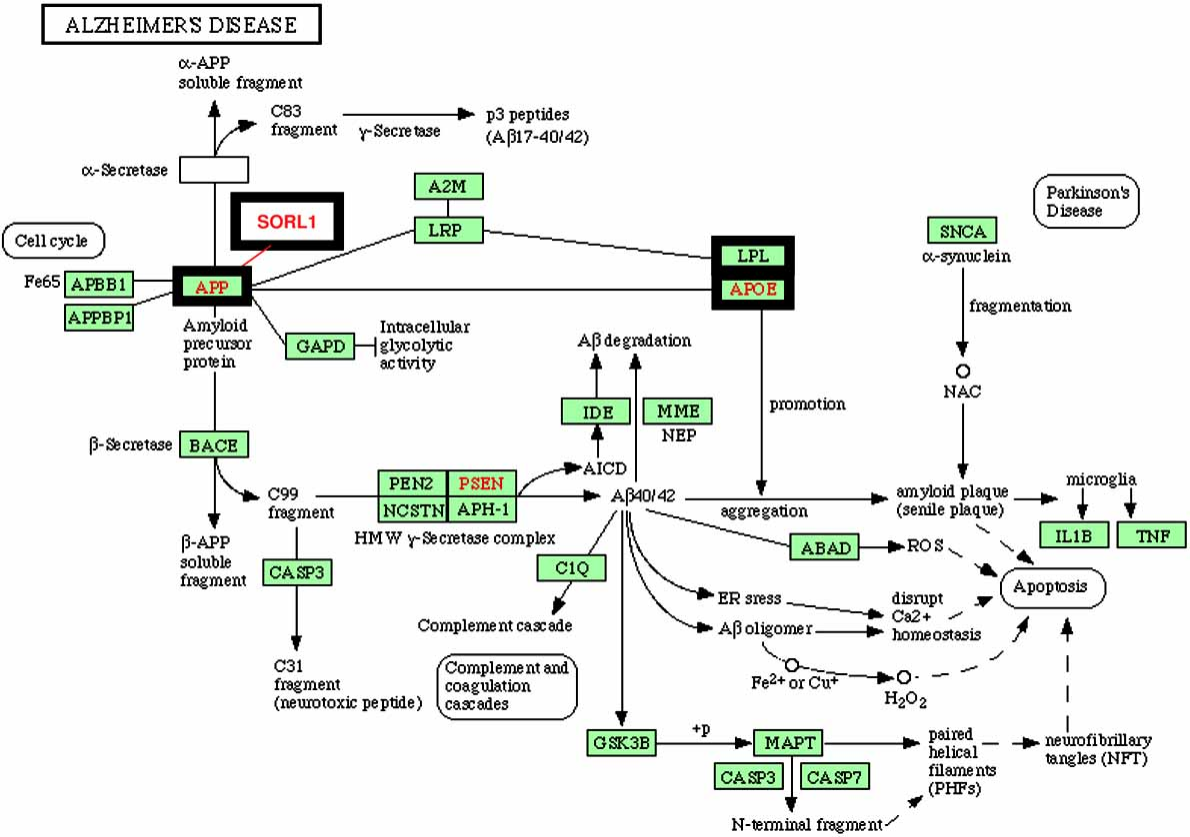
**Common polymorphisms in the genes associated with Alzheimer’s Disease (AD)**. The pathway diagram is from KEGG (http://www.genome.jp/kegg/). Three common mutations (SORL1, LRP, APOE) interact with each other and with APP. The outcome of such interactions in the genomic background of a single individual is difficult to predict.

**Fig. (3):**
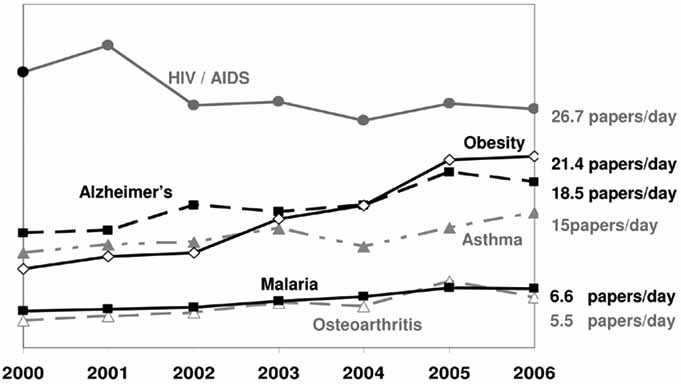
**Average number of papers published per day for some common diseases**. The numbers given on the right hand side of the figure are the 2006 averages. The numbers were collected from the Web of Science (http://scientific.thomson.com/products/wos/) for the period 1^st^ January 2000 to 1^st^ January 2007 by searching for papers with the disease name in their keyword listing.

**Fig. (4):**
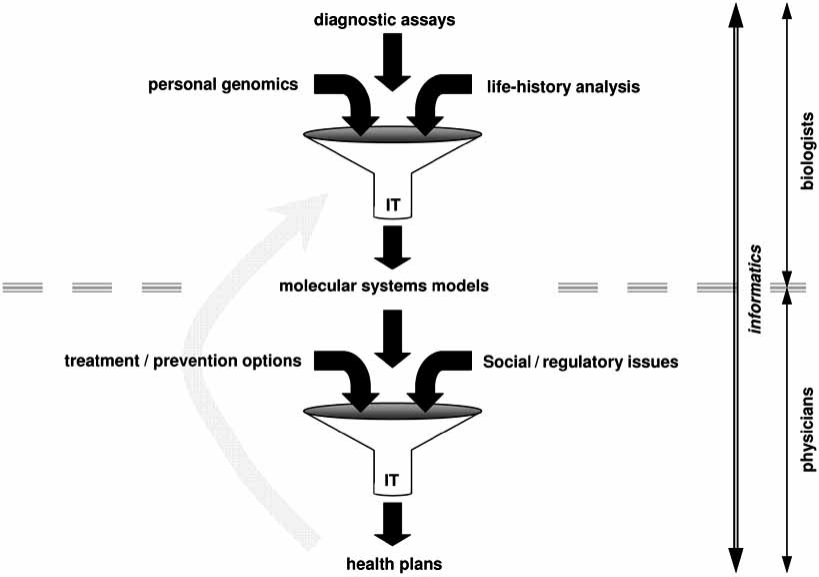
**Proposed integration of molecular systems biology computational tools with medical informatics decision-support tools**. The cartoon funnels represent information-theoretic frameworks for aggregation, filtering and summary-reporting of local test results with general background knowledge mined from the literature and 3^rd^ party databases. IT stands for Information Technology. As described in the text, all data processing is assumed to be performed through security-certified web services. All data will reside with the patient and be available only for patient-authorized appropriately anonymized analysis tasks. In this way, the scheme provides a mechanism for data security while also allowing researchers to analyze and learn from patient data. Although not shown explicitly in the figure, the proposed integration will also facilitate understanding of personal data by patients through enabling a large variety of software tools to explore the same data using a shared software infrastructure.
